# Empirical Evaluation of Oligonucleotide Probe Selection for DNA Microarrays

**DOI:** 10.1371/journal.pone.0009921

**Published:** 2010-03-29

**Authors:** Jennifer G. Mulle, Viren C. Patel, Stephen T. Warren, Madhuri R. Hegde, David J. Cutler, Michael E. Zwick

**Affiliations:** Department of Human Genetics, Emory University School of Medicine, Atlanta, Georgia, United States of America; Ohio State University Medical Center, United States of America

## Abstract

DNA-based microarrays are increasingly central to biomedical research. Selecting oligonucleotide sequences that will behave consistently across experiments is essential to the design, production and performance of DNA microarrays. Here our aim was to improve on probe design parameters by empirically and systematically evaluating probe performance in a multivariate context. We used experimental data from 19 array CGH hybridizations to assess the probe performance of 385,474 probes tiled in the *Duchenne muscular dystrophy* (*DMD*) region of the X chromosome. Our results demonstrate that probe melting temperature, single nucleotide polymorphisms (SNPs), and homocytosine motifs all have a strong effect on probe behavior. These findings, when incorporated into future microarray probe selection algorithms, may improve microarray performance for a wide variety of applications.

## Introduction

DNA-based microarrays have become central to current biomedical research for a host of diverse applications[1], ranging from assessment of genomic copy number (array CGH)[2], [3], [4] and identification of transcription binding sites (ChIP-chip)[5], [6], [7] to resequencing[8], [9], [10], [11], [12] and SNP genotyping[13], [14], [15], [16], [17]. Of great excitement is a recent application, microarray-based genomic selection (MGS), which can serve as a bridge to next-generation resequencing technologies, enabling the complete ascertainment of sequence variation in large, specific regions of the human genome[18], [19], [20]. Appropriate microarray design is fundamental to the success of these experiments.

To optimize the design, production, and performance of DNA microarrays, selecting the right oligonucleotide sequences to be tiled is essential. Probes should be chosen to maximize the information contributed by every available feature on an array. What constitutes “information” on a DNA microarray is largely the effective binding of a probe to its target sequence, in the absence of cross-hybridization of non-target sequence; this is a universally desirable property, regardless of the microarray application. There are numerous algorithms based on various criteria to aid in the selection of probe sequences. Bertone and colleagues focused on optimizing methods for choosing probes in a genomic region heterogeneous for repetitive and unique elements, with the aim of maximizing the percentage of non-repeat bases covered[21]. Graf and coworkers expanded on these results by developing a probe uniqueness score (*U*) based on the number of unique substrings of sequence within a given target region[22]. This group developed a probe-selecting algorithm incorporating *U*, melting temperature (Tm), and synthesis cycle number with sequence-specific filters. With this algorithm they have demonstrated acceptable coverage of the mouse genome[22]. Array manufacturers have used similar methods to create proprietary, platform-specific algorithms for array design. For example, Roche NimbleGen (Madison, WI) combines synthesis cycles (with a sliding upper limit depending on final probe length), Tm, repetitive element exclusion, and uniqueness measures to select probes[23]. Nonetheless, the probes selected by all these algorithms have yet to be evaluated experimentally. Moreover, such direct empirical evaluation of probe performance would drive better design algorithms.

There has been some empirical characterization of probe behavior by various groups. Sharp et al. characterized the performance of Roche NimbleGen probes in detail using an array CGH format. Seven individuals with a validated genomic imbalance on chromosome 15q, where each individual has from one to six copies of the same locus, were assessed on a custom-designed array with 91,069 probes available to detect copy number[24]. The outcome measure was the Pearson's correlation coefficient for a given probe (r) between normalized log(2) ratio values and copy number across experiments. This group found that probe uniqueness, SNP content, probe length, Tm, and guanine homopolymers all influenced probe performance[24]. The negative influence of guanine- or purine-rich sequence has been noted by other groups[25], [26]. However, all the variables considered by Sharp et al. were examined individually, without a multivariate analysis that could enable detection of correlated variance components. An additional univariate study found probe uniqueness and homopolymer presence (> length 5), but not probe length or Tm, to affect the resolving power of array CGH to detect deletions in both human and *C. elegans* experiments[27]. As with Sharp et al., a multivariate analysis was not performed.

We sought to refine probe design parameters by evaluating probe performance in a multivariate context empirically and systematically. We chose to focus on Roche NimbleGen arrays, since this format is both the most amenable to custom design and currently has the highest probe densities. In most analysis techniques, data for a given probe is the log(2) ratio of signal intensity between two samples. We reasoned that well-behaved probes ought to give consistent log(2) ratios near 0, whereas poorly behaved probes will vary around 0 more widely. Therefore we elected to focus on the variance in the log(2) ratios across multiple experiments as a measure of poor probe behavior. We believe this measure simultaneously identifies probes with both unreliable binding by target sequences as well as low target capture, since low signal often inflates the log(2) variance. We used experimental data from 19 array CGH hybridizations to evaluate probe performance for 385,474 probes tiled in the *Duchenne muscular dystrophy* (*DMD*) region of the X chromosome. We used as predictor variables several sequence-derived probe characteristics, including Tm, probe length, probe GC content, probe purine content, and the presence of a known SNP in the probe. We also explored whether the presence and length of homoadenine, homocytosine, homoguanine, or homothymidine sequence motifs could influence probe performance. The outcome measure, variance in normalized log(2) ratios across experiments, was dichotomized based on the observed distribution of values across probes. Following univariate analysis, we incorporated multiple predictor variables into hierarchical models to reveal the subset of predictor variables with the effects on probe performance. Our results indicate that Tm, the presence of a SNP, and the presence of homocytosine motifs all influence probe behavior. These data should improve array design substantially by refining the algorithms to optimize probe performance.

## Results

**Figure 1:**
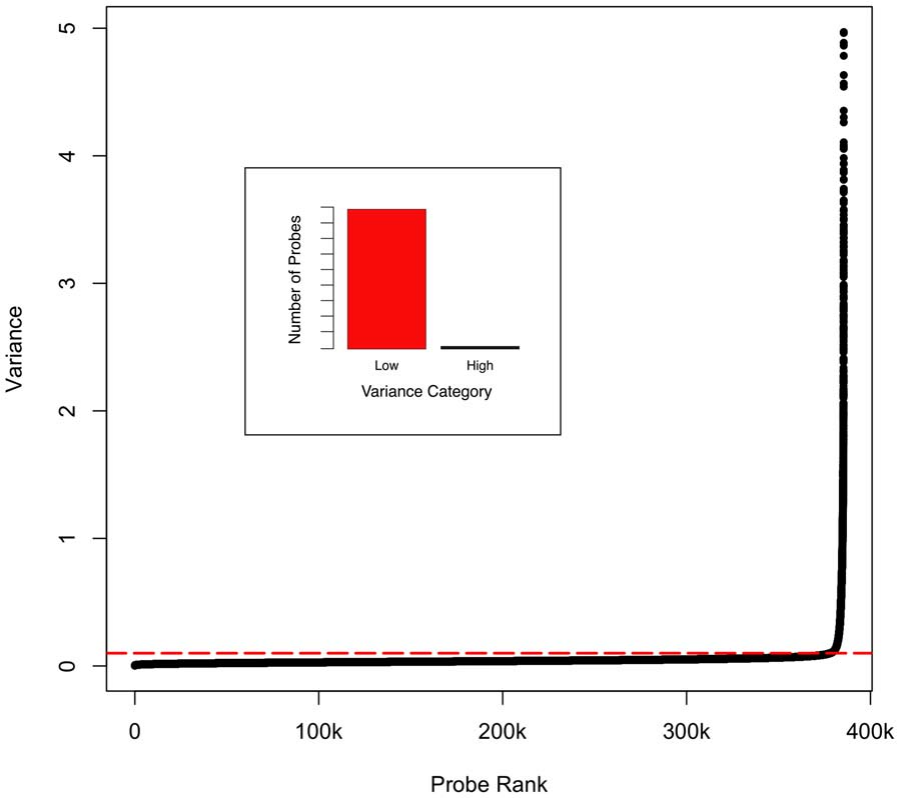
Observed variance in probe performance across multiple array CGH experiments. 384,475 probes are ranked by variance in normalized log(2) ratios across 19 array CGH experiments. Rank order of probe is plotted on the x-axis, variance on the y-axis. A dotted horizontal line is drawn at variance  = 0.1, where probes are dichotomized according to “low” or “high” variance. Inset: number of probes that fall into each category.

**Figure 2:**
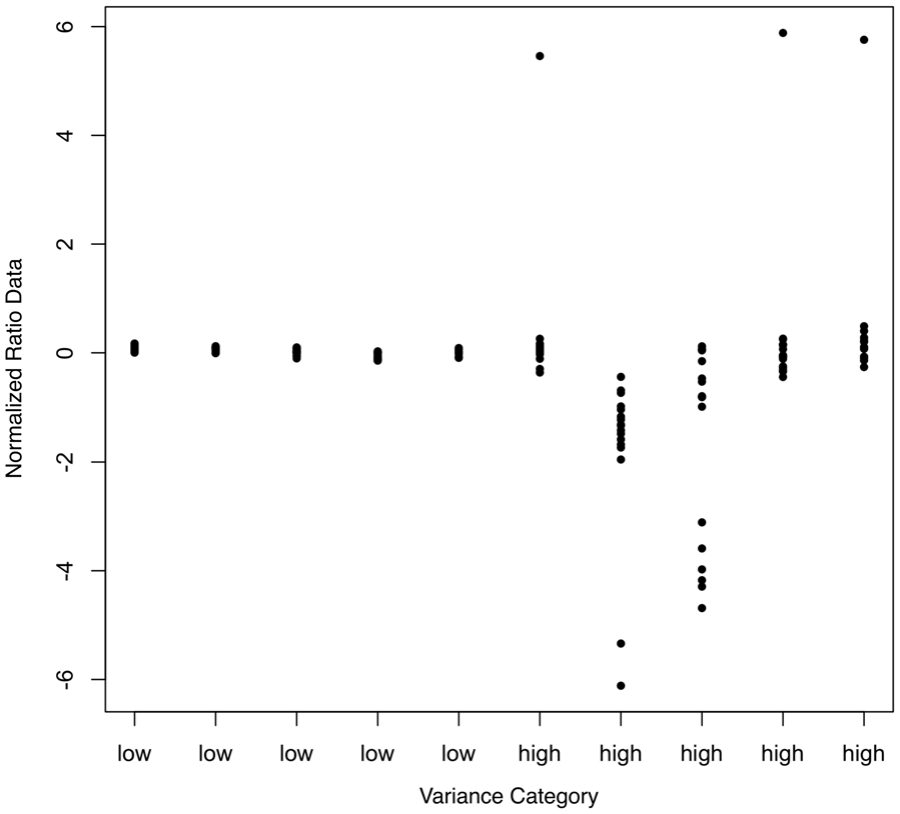
Examples of data from low- and high-variance probes. Normalized log(2) ratio data for 5 probes of low variance and 5 probes of high variance across multiple experiments. High-variance probes are likely to have outlier values for one or more experiments, but also have large variance even when outliers are excluded.

Examining the distribution of variance revealed that the majority of probes have low variance (<0.1, n = 378,057), with only ∼2% of probes showing high variance (>0.1 and <4.97, n = 7,417). We dichotomized the probes based on a cutoff of 0.1 (Figure 1, and see Figure 2 for raw data examples). Results of our univariate analysis are described in Tables 1, 2 and 3. Variables that were found to be highly significant (p<0.01) included Tm, probe length, GC content, presence of a SNP, average heterozygosity of a SNP, polyA, polyC, polyG, and polyT. Low-variance probes had a higher Tm, shorter length, higher GC content, shorter polyA or polyT runs, and longer polyC or polyG runs. Low-variance probes were also less likely to have a SNP. Among those probes that did have a SNP, low-variance probes have less common (i.e., lower frequency) SNPs. Purine content was not associated with probe performance.

**Table 1:** Results of univariate analysis, quantitative variables.

	Low Variance Probes	High Variance Probes	p-value
**N**	378,058	7417	
**Variance**	0.039	0.379	
**Tm**	65.13	63.87	<2.2e-16
**Length**	56.55	56.97	<2.2e-16
**GC content (%)**	35.93	33.28	<2.2e-16
**Purine content (%)**	50.47	50.56	0.4538
**Proportion with SNP**	0.141	0.158	.000011
**SNP heterozygosity**	0.144	0.157	0.032

**Table 2:** Percent of low and high variance probes with homopolymer runs (nucleotide, variance).

Size of Homopolymer	A, Low	A, High	T, Low	T, High	C, Low	C, High	G, Low	G, High
**1 or 2**	21.3	18.0	23.8	20.6	73.7	80.1	74.0	77.5
**3**	37.3	37.8	38.0	37.9	23.1	18.0	22.8	20.2
**4**	27.8	30.3	26.2	28.4	3.2	1.9	3.2	2.3
**5**	11.5	12.2	10.3	11.8	na	na	na	na
**6**	1.8	1.5	1.5	1.0	na	na	na	na
**7**	0.3	0.2	0.3	0.3	na	na	na	na

**Table 3:** Results of univariate analysis, categorical variables.

	A	C	G	T
**chi-square**	62.79	69.70	160.97	51.36
**p-value**	3.213e-12	1.182e-13	<2.2e-16	7.021e-12

Tm, probe length, and GC content all appear to be related to one another. To examine the degree of relationship, we computed Pearson's correlations among these variables. This revealed a highly correlated structure among these three variables (Table 4). To discern which variable(s) were having the primary effect on probe performance, we compared Akaike's information criterion (AIC) from single-term models, where probe performance was regressed on length, GC content, or Tm (Table 5). Based on the AIC, Tm is the predictor that best fits the data. We then compared two-term models with Tm and either length or GC content to assess whether there were residual effects of these variables after accounting for Tm. When either length or GC content is included in a model with Tm, neither length nor GC content is a significant predictor of probe performance. Furthermore, based on AIC values, using Tm alone is the most parsimonious model that fits the data best (Table 6). This implies either that Tm is the primary factor among these variables contributing to probe performance, or that our Tm calculation effectively describes length and/or GC content.

**Table 4:** Pearson's correlations between predictor variables (all p <2.2e-16).

	Tm	Length
**GC content**	0.966	−0.466
**Length**	−0.397	

**Table 5:** Comparison of single-term models with Tm, GC content, and probe length.

Single-Term Models	AIC
**Tm**	72273*
**GC content**	72333
**Length**	73231

**Table 6:** Testing for residual effects of GC content or probe length, after adjusting for Tm.

Model Includes	Estimates	beta	se	p-value	Model AIC
Tm	Tm	−0.111	0.003	<2e-16	72273
Tm + GC content	Tm	−0.108	0.014	8.89e-15	72275
	GC content	−0.002	0.006	0.794	
Tm + length	Tm	−0.113	0.003	<2e-16	72273
	Length	−0.005	0.003	0.126	

We then examined the potential association of the remaining significant variables (polyA, polyT, polyG, polyC, presence of a SNP) with probe performance by adding these terms to a model including Tm. The AIC of each model shows that the presence of a SNP and polyC are independently predictive of probe performance, but the remaining variables (polyA, polyT, polyG) do not have a significant relationship to probe performance after adjusting for Tm (Table 7). The presence of a SNP is significantly associated with poor probe performance, whereas the presence of poly-cytosine motifs are predictive of good probe performance, after adjusting for Tm effects. The majority of this latter effect is contributed by the tricytosine motif, with a minority contributed by a quadcytosine motif. Five or more cytosines in a row appear to have no effect on probe performance (Table 8), although this is likely a reflection of the small number of such observations.

**Table 7:** Comparison of models with remaining predictor variables.

Model With Tm and:	AIC
**SNP**	72255
**PolyA**	72268
**PolyC**	72253
**PolyG**	72276
**PolyT**	72270
**PolyC + SNP**	72236

**Table 8:** Final model including Tm, SNP, and polyC.

Variable	beta	se	p-value	Final Model AIC
Tm	−0.107	0.004	<2e-16	72236
SNP	0.143	0.032	8.32e-06	
Poly C (3)	−0.134	0.031	1.81e-05	
Poly C (4)	−0.204	0.087	0.0185	
PolyC (5)	−9.15	54.5	0.8667	

## Discussion

We reasoned that the log(2) ratio from a given probe in an array CGH experiment is a useful proxy for the information yielded by that probe. We propose that the variance in the log(2) ratio across multiple experiments captures poor probe behavior, such as unreliable binding by target sequences as well as low target capture, since at low signals the log(2) variance is often inflated. We explored other measures of probe performance from our array CGH data, including raw intensity measures for Cy3 (532 channel) and Cy 5 (635 channel) both separately and combined, and the ratio of the standard deviation to the mean intensity across experiments for Cy3, Cy5, and combined intensities. These measures all yielded very similar distributions, with most probes behaving in a consistent fashion, but with ∼2% of probes giving highly unreliable data. This implies that we have identified a true set of poorly performing probes.

Using this definition of poorly performing probes, our study demonstrates that Tm, homocytosine motifs, and the presence of a SNP are all significant predictors of probe performance in microarray design. Tm is a highly significant predictor: an increase in Tm of ten degrees renders a probe almost three times more likely to be in the low-variance (i.e., higher reliability) category. The next largest predictor of probe performance is presence of a SNP. After adjusting for Tm, we find there is a 15% increase in the likelihood that a probe will be in the high-variance (i.e., lower reliability) category if it contains a known SNP. Further, we find that homocytosine motifs of size 3 or greater are associated with reliable probe performance.

The array we used is specific to the *DMD* region of the X chromosome. An advantage to this experimental design is that patient samples are unlikely to have additional copy number variants, aside from the pathological deletions and duplications already identified and excluded prior to this analysis. This is important, because if there were additional deletions or duplications, the variance of the log(2) ratio data would be artificially inflated, and we would run the risk of misclassifying probes, leading our analysis astray.

Our analysis identifies poorly behaving probes as those that do not behave reliably across multiple experiments. There is another potential class of poorly performing probes: those that have saturated intensity due to signal from cross-hybridization of non-unique sequence. These probes might behave consistently from experiment to experiment, qualifying as well-behaved according to our criteria. However, because potential probes are first screened for non-unique sequence, and probes with too many genomic matches discarded, this is likely a small source of misclassification.

Ultimately, our results are in predominant agreement with other analyses, in particular those of Sharp et al. As in previous reports, we find SNP content and Tm to be significant predictors of poorly performing probes. However, because our analysis could be extended to a multivariate format, we were able to test directly among the related variables Tm, GC content, and probe length to arrive at the predictor variable(s) with the largest effect(s). While Sharp et al. attempted to correct for relationships among predictor variables, they did so in an indirect manner, measuring covariance between variables, and their analysis framework did not allow simultaneous adjustment for multiple predictor variables. Furthermore, Sharp et al. used an approximation for Tm that did not take into account the microarray environment. The Tm calculation we have used is one most appropriate for surface-bound oligonucleotides, and may more accurately estimate the true melting temperature of the oligos. Nevertheless, it should be noted that the Sharp et al array was specific to a single region of the genome, on chromosome 15. It is possible that their results are specific to this single genomic interval and may not be generalized across the genome, which perhaps accounts for the minor differences between our results and those of previously published studies.

There are likely two dimensions to probe performance: the ability of a probe to be correctly synthesized on an array, and the ability of a correctly synthesized probe to bind its target. The variables found to have the strongest effects in this analysis, Tm and presence of a SNP, are likely related to hybridization and not synthesis; however, the contribution of the tricytosine and quadcytosine motifs to probe performance remains unclear. It is possible that three or four cytosines in a row allow a probe to assume a three-dimensional conformation that renders it highly available to its target sequence. It is also possible that, once a target sequence binds to a probe with more than three cytosines, it is stably bound due to the large number of hydrogen bonds holding it in place. Another possibility is that during synthesis, cytosines have a slightly higher coupling efficiency than the three other nucleotides, and therefore three or more cytosines in a row are the motifs likely to have the fewest base misincorporations during array manufacture.

Sharp et al. have shown an excess of polyG motifs in poorly performing probes on Roche NimbleGen arrays, the same technology used in our study[24]. A second group has previously shown that on Affymetrix resequencing arrays, purine-rich (and specifically guanine-rich) probes are overrepresented among probe failures[24], [25]. Our analysis fails to discern any relationship between purine-rich sequences or polyG motifs and poor probe performance. Results from our univariate analysis imply that there is an overabundance of polyG motifs in low-variance (or “good”) probes. However, when we incorporate Tm and polyG motifs in a model together, there is no significant effect from the polyG motifs. It is therefore possible that prior reports of polyG sequences associated with poor probe performance actually reflect a Tm relationship. In fact, Sharp et al. show that polyG is positively correlated with Tm, but speculate that polyG motifs have an effect independent of Tm[24]. They do not show this directly, however, as it was not possible to test both variables simultaneously in their analysis framework. Furthermore, Zwick et al. and Cutler et al. also do not test for Tm[25], [26]. It therefore remains possible that the polyG relationship really reflects a Tm relationship best accounted for when we include Tm in a multivariate analysis. It is also possible that polyG has different effects depending on the genomic region being examined (or on the organism, as Zwick et al. were sequencing *Bacillus anthracis*). The genomic region on 15q11 tested by Sharp et al. is 41% GC, compared with 36.3% GC in the *DMD* region we tested in our study, suggesting that GC content alone is not responsible for this discrepancy. Alternatively, it is possible that manufacturers have already responded to known purine (or guanine) issues by improving their synthesis chemistries in the interval between the two studies.

**Figure 3:**
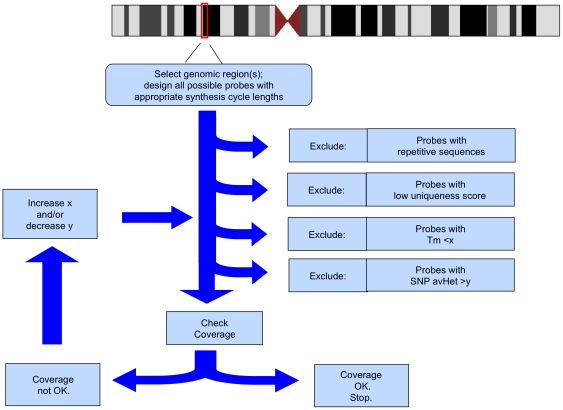
Proposed algorithm to refine probe selection.

Our analysis here examines probes already selected by the manufacturer's algorithm. Such algorithms actually work well, as evidenced by the fact that fewer than 2% of the probes on our array had high variance across experiments. However, the analysis we present here allows for a quantifiable estimate attached to each predictor variable, providing some much-needed guidance when selecting probes for custom-designed arrays. For example, although it was already known that both Tm and SNP are important, our analysis implies that low Tm is “worse” than the presence of a SNP in a probe. We propose a sequential series of considerations when designing probes that could significantly reduce probe failure (Figure 3). Such enhanced probe selection, however modest, could still have a major experimental impact (Figure S1). As microarray probe density continues to increase, a 2% probe failure rate represents a substantial cost in data loss. By way of illustration, with Roche NimbleGen's latest high-density array of 2.1 million probes, a 2% rate translates to the failure of 42,000 probes. What this means for array CGH is that such losses could obscure copy number discrimination or reduce precision in breakpoint determination. For MGS, critical genomic regions targeted for capture and resequencing may be missed. Thus we propose that the data reported here will help reduce probe failure and allow for maximum data extraction from microarray experiments.

## Materials and Methods

### Experimental data

We obtained 19 de-identified patient DNA samples with previously characterized *DMD* gene structural mutations (deletions and duplications) from the Emory Genetics Laboratory, OHSU DNA Diagnostic Laboratory, and LabPLUS, Auckland, New Zealand. All samples were stripped of personal identifiers and numbered randomly. This study was approved by the Emory University Institutional Review Board (#IRB00024817).

All patients had prior clinical validation of *DMD* structural mutations identified by multiplex PCR of 32 exons and/or Southern blot. We evaluated DNA from these patients with array CGH using a custom-designed array with 385,474 probes in the *dystrophin* gene region, which spans 2,222,000 bases on chromosome X (31,046,000–33,268,000; www.ucsc.edu). The vast number of probes permits oversampling of the region; the average spacing between probe starts is less than six bases. The array used in these experiments was designed and manufactured by Roche NimbleGen Systems, Inc. (Madison, WI). Roche NimbleGen used in-house design criteria to select probes. These design criteria included four main components: (1) an upper limit on synthesis cycles, (2) probe selection based on Tm, (3) avoidance of repetitive elements, and (4) a proprietary “uniqueness measure”[23].

DNA extraction was performed on patient DNA using the Gentra Systems Puregene DNA extraction kit according to the manufacturer's instructions. Normal male and female reference DNA was obtained from Promega, Inc. Each patient and reference DNA sample was sonicated such that fragment size was between 500–2000 bases, as verified on a 1% agarose gel. Patient and reference DNA samples were then labeled using Klenow enzyme (NEB) and Cy3 or Cy5 9 mer wobble primers (TriLink Technologies), respectively. After labeling, each sample was purified by isopropanol precipitation and reconstituted in ultra-pure water. We combined 13 ug each of labeled patient and reference DNA, and desiccated the mixture in a Vacufuge (Savant DNA 120), then resuspended in appropriate hybridization buffer along with Cy3 and Cy5 control CPK6 50 mer oligonucleotides. This mixture was hybridized to the array for 16–20 h at 42°C in a Maui Hybridization instrument (BioMicro Systems). Arrays were then washed according to the manufacturer's recommendation and immediately scanned on a GenePix 4000 scanner (Molecular Devices). After scanning, intensity data were extracted from images, and within-array normalization was accomplished using manufacturer-provided software (NimbleScan). Normalized log(2) ratio data were analyzed using the GLAD[28] program as implemented in R.

### Predictor variables

Characteristics we hypothesized might be related to probe performance were: probe melting temperature (Tm), probe length, probe GC content, probe purine (AG) content, the presence/absence and average heterozygosity of a known SNP, maximum size homoadenosine sequence, maximum size homocytosine sequence, maximum homoguanine sequence, and maximum homothymidine sequence in a probe. These variables were derived from the probe sequence via a custom Perl program.

Tm was calculated using the thermodynamic model proposed by Vainrub and Pettitt[29], which modifies the Langmuir isotherm to appropriately account for electrostatic interactions among surface-bound molecules, which are present in microarray environments, as follows:
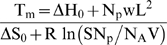



Briefly, let ΔH_0_ be the probe reference state enthalpy, ΔS_0_ the probe reference state entropy, N_p_ the probe density, *w* the interaction strength constant, L the probe length, R the universal gas constant, S the feature size surface area, V the hybridization volume, and N_A_ Avogadro's number. Entropy, enthalpy, and initiation parameter values for calculating ΔH_0_ and ΔS_0_ were obtained from the unified nearest-neighbor thermodynamic method proposed by SantaLucia[30]. We assumed values of 50,000 per square micron for N_p_, 4×10^−16^ for *w*, 20×24 *u*M for S, and 2.0e-4 for V. All values are approximately consistent with our experimental conditions. Tm was calculated according to the formula above from the sequence composition of each probe, as implemented in a custom Perl program.

Probe length was calculated based on the probe sequence (range: 50 to 75 nucleotides). GC content and purine content were expressed as proportions. The presence of a known SNP was assessed by obtaining all known SNP positions cataloged in dbSNP (build 128) within the 2.22-Mb *DMD* region on chromosome X (n = 6883). We asked whether any SNPs mapped within the interval defined by the probe start and stop positions for all probes on the array; probes with SNPs were coded as “1”, and those without were coded as “0”. For probes with SNPs, we recorded the type of SNP and, when available, the average heterozygosity. For the maximum homonucleotide sequence within a probe, we recorded the longest stretch of homonucleotides >2 for all four possible nucleotides (A,G,C,T; four different variables for each probe: polyA, polyC, polyG, polyT). Probes with a maximum 1 or 2 of any homo[A,G,C,T] sequence were coded as “0”, probes with 3 homo[A,G,C,T] were coded “1”, probes with 4 homo[A,G,C,T] were coded “2”, and so on. When tabulating the data, we noticed that for long homopolymer runs (>7 for A and T; >4 for C and G) there were cells with n<5. Small numbers in any one cell of a table can inflate a chi-square test, but our sample size renders Fisher's exact test computationally intractable. As a compromise, we chose to collapse the highest categories downward, until all cells had a minimum value of 5, as follows: for A and T, probes with runs 7 or greater (maximum run size  = 9 for A, 8 for T) were collapsed into a single group (number of recoded probes  = 138 for A, n = 89 for T); for G and C, probes with runs 4 or greater (maximum run size  = 6 for C, 8 for G) were collapsed into a single group (number of recoded probes  = 96 for C, n = 175 for G).

### Outcome variable

For each array, we excluded probes within the *DMD* region that were known to be deleted or duplicated for each patient; we kept probe data only for regions with equal genomic content between test and reference. To avoid unfairly inflating the variance in probes because of uncertainty in the predicted breakpoints of known copy number variants, we also excluded probes within 25 kb of a predicted breakpoint. For all 385,474 probes, we calculated the variance of the normalized log(2) ratio across all experiments (range 0.0018–4.97). Examining the distribution of variance revealed that the majority of probes have low variance (<0.1, n = 378,057), with only a fraction of probes displaying high variance (>0.1 and <4.97, n = 7417). We dichotomized the probes based on a 0.1 variance cutoff.

For univariate analysis, we compared the distribution of predictor variables between low- and high-variance probes using Student's t-test for continuous variables and a chi-square test for categorical variables. To examine bivariate relationships among Tm, GC content, and probe length, we computed Pearson's correlations among these variables. After univariate exploration of the data, variables with significant differences (p<0.05) between low- and high-variance probes were investigated further in multivariate analysis using a logistic regression model. To best capture the nonlinear relationship between homonucleotide runs and probe performance, dummy categorical variables were created. Dummy categorical variables were also created for Tm, in 5-degree bins, to confirm that the Tm-probe performance relationship was linear across the Tm range (data not shown). Models were compared using Akaike's information criterion (AIC)[31].

## Supporting Information

Figure S1 Effect of Removing Bad Probes. Array CGH data for 5 samples. Normalized log(2) ratio plotted by position. Top panel includes data for all probes. Bottom panel includes data for 98% of the data (excludes 2% of probes with excessive variance).
(0.23 MB PDF)
