# Supplementary material for: Empirical Evaluation of Oligonucleotide Probe Selection for DNA Microarrays
Source: PLoS One. 2010 Mar 29;5(3):e9921. doi: 10.1371/journal.pone.0009921 (PMC2847945; doi:10.1371/journal.pone.0009921)
Supplement: Figure S1 — Effect of Removing Bad Probes. Array CGH data for 5 samples. Normalized log(2) ratio plotted by position. Top panel includes data for all probes. Bottom panel includes data for 98% of the data (excludes 2% of probes with excessive variance). (0.23 MB PDF) [file pone.0009921.s001.pdf]

Additional File 1: **Effect of Removing Bad Probes**. Array CGH data for 5 samples. Normalized log(2) ratio plotted by position. Top panel includes data for all probes. Bottom panel includes data for 98% of the data (excludes 2% of probes with excessive variance).

Sample DMDOSU1, all data

Sample DMDOSU1, high variance probes removed

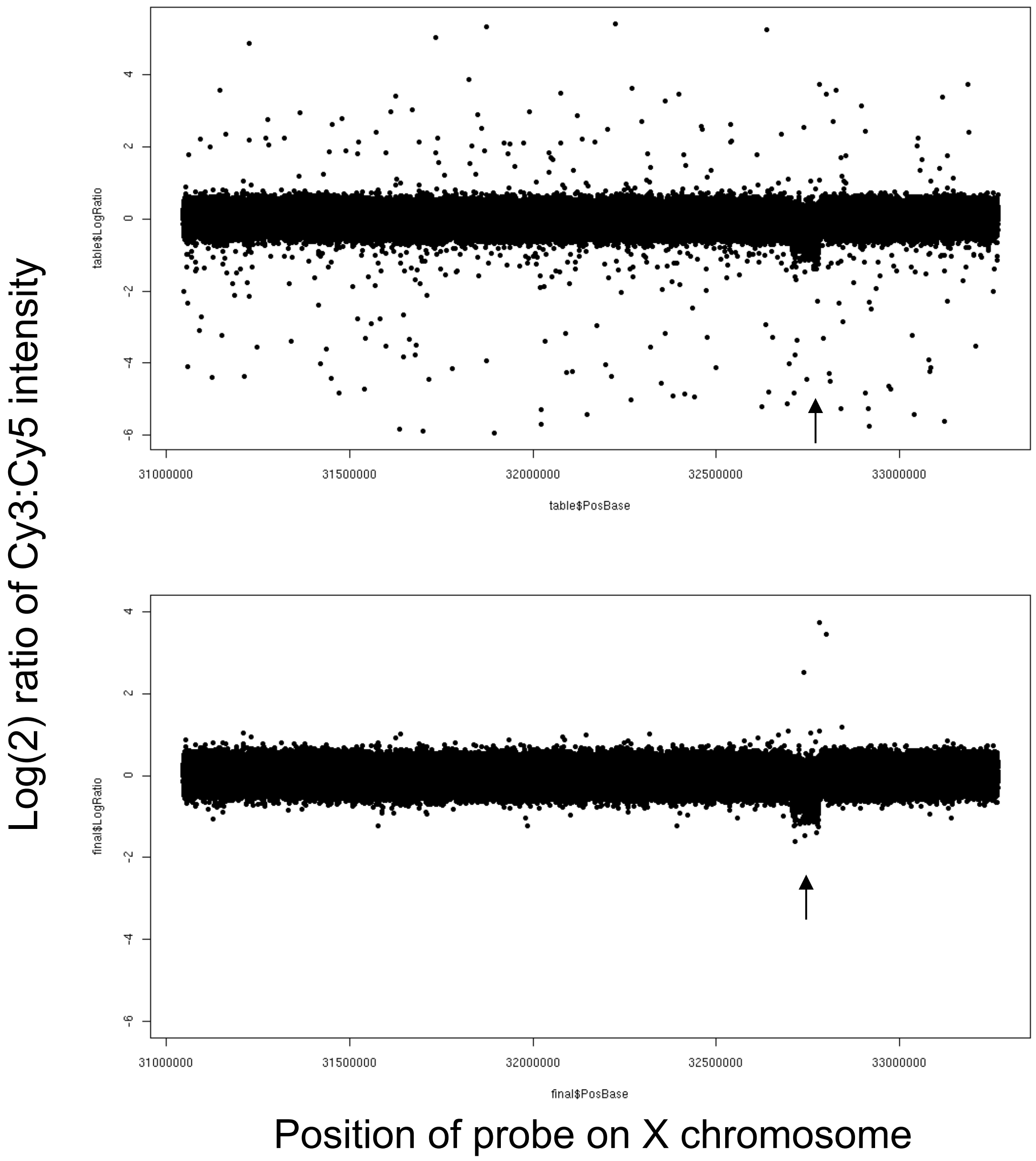

Sample DMDOSU5, all data

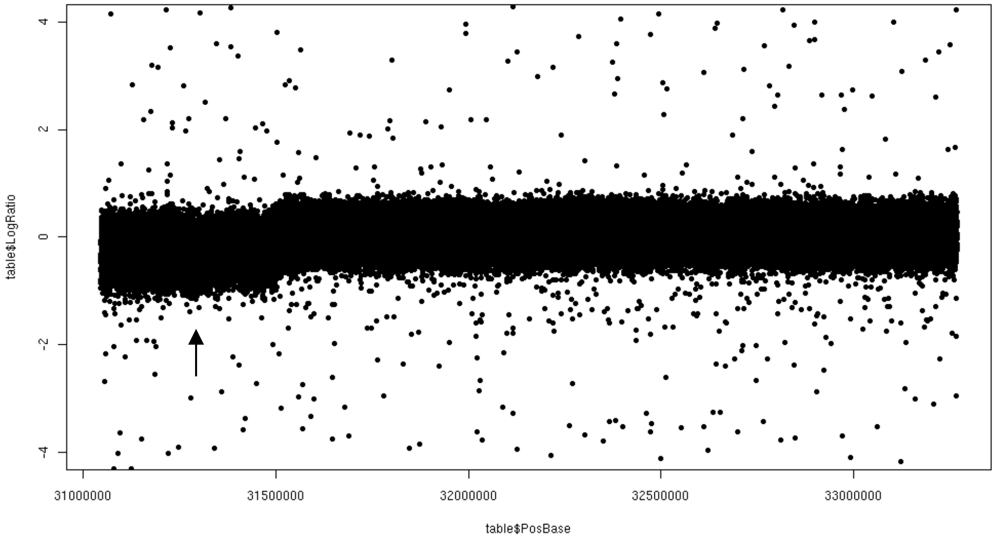

Sample DMDOSU5, high variance probes removed

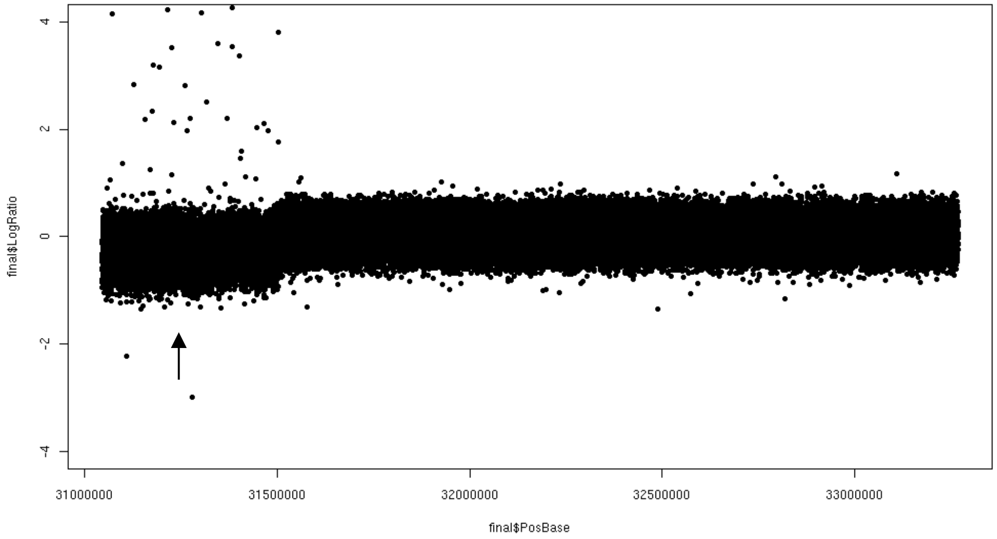

Log(2) ratio of Cy3:Cy5 intensity

Position of probe on X chromosome

Sample DMDOSU12, all data

Sample DMDOSU12, high variance probes removed

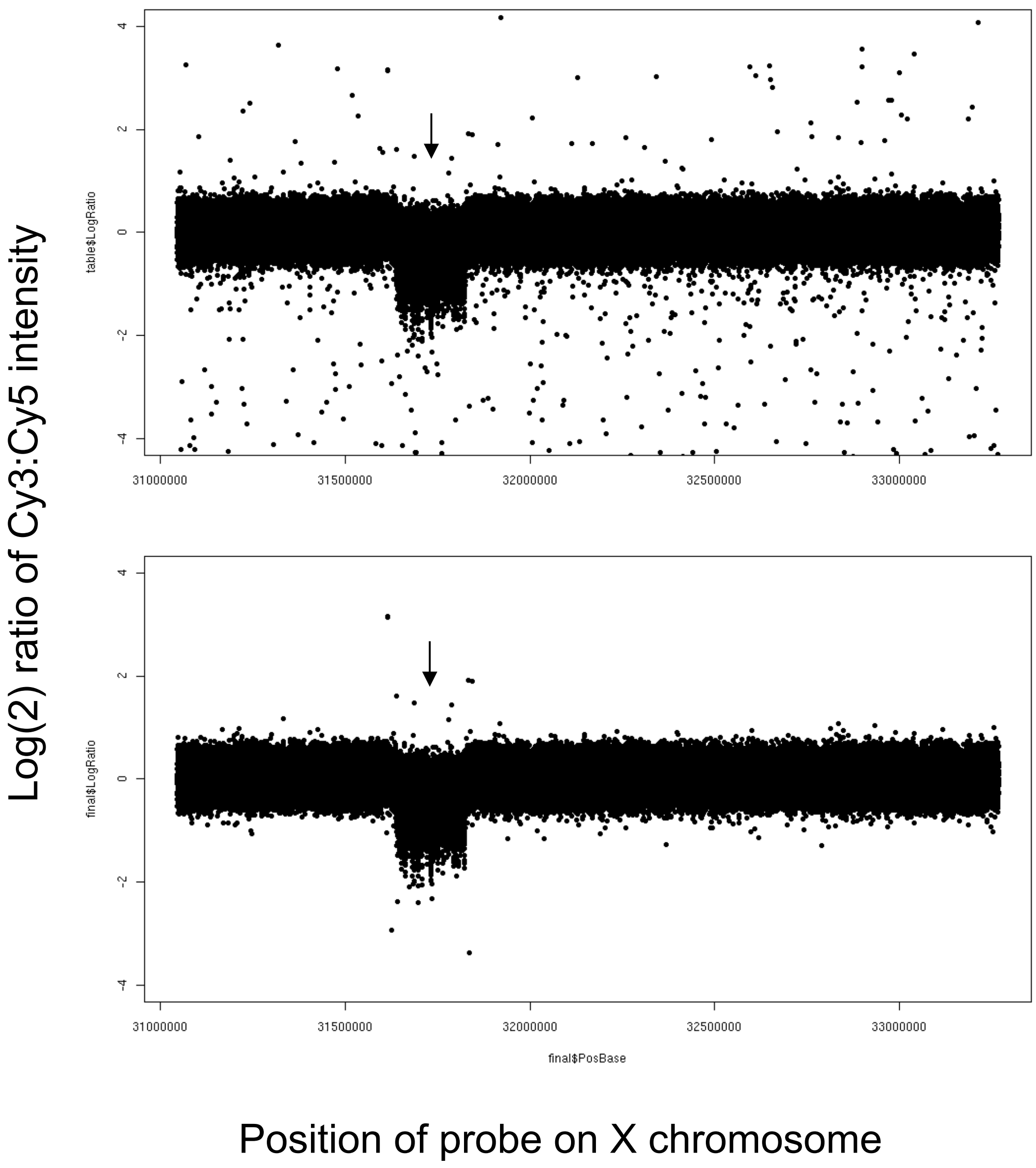

Sample DMDOSU11, all data

Sample DMDOSU11, high variance probes removed

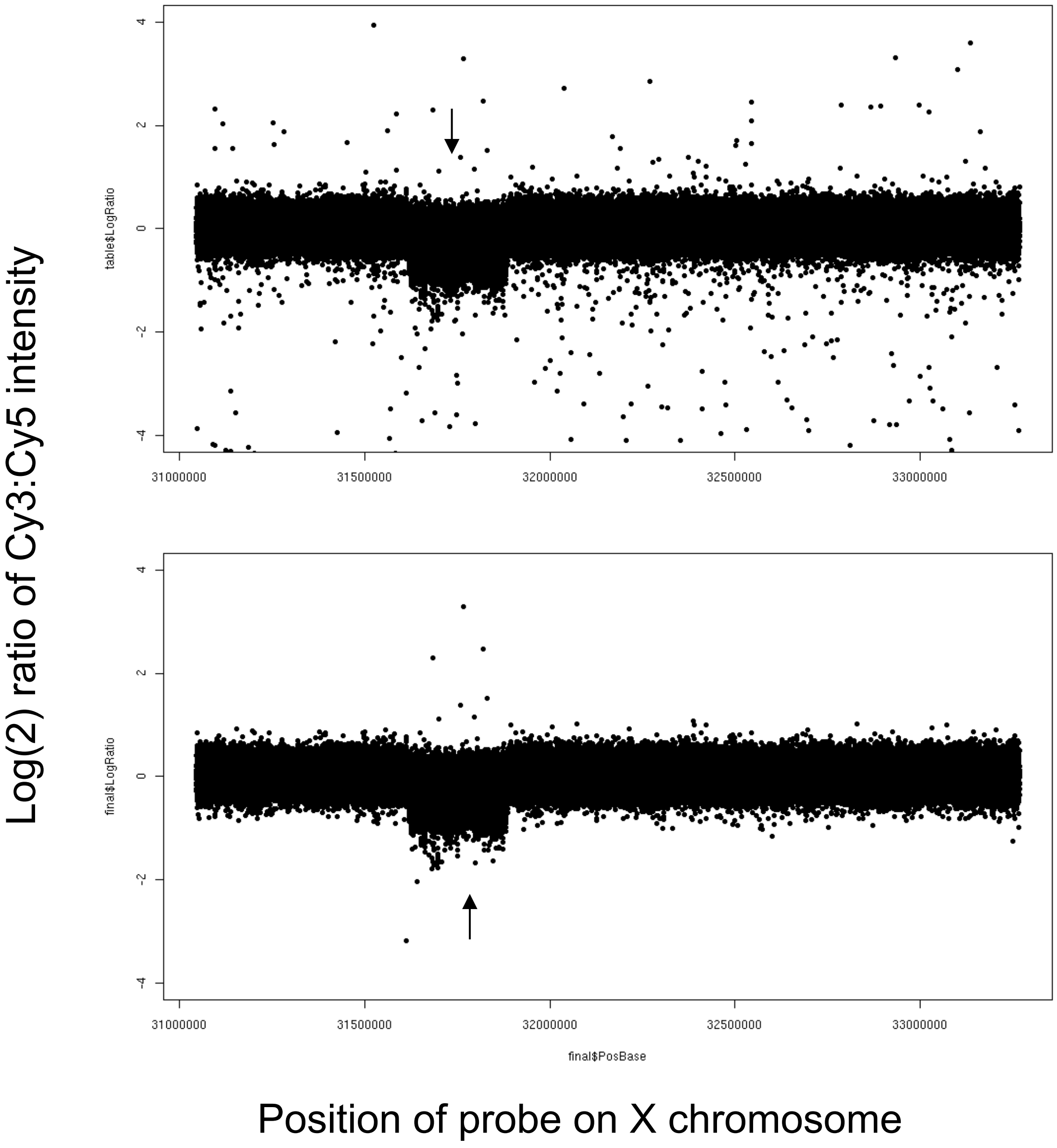

Sample 15685,
all data

Sample 15685,
high variance
probes removed

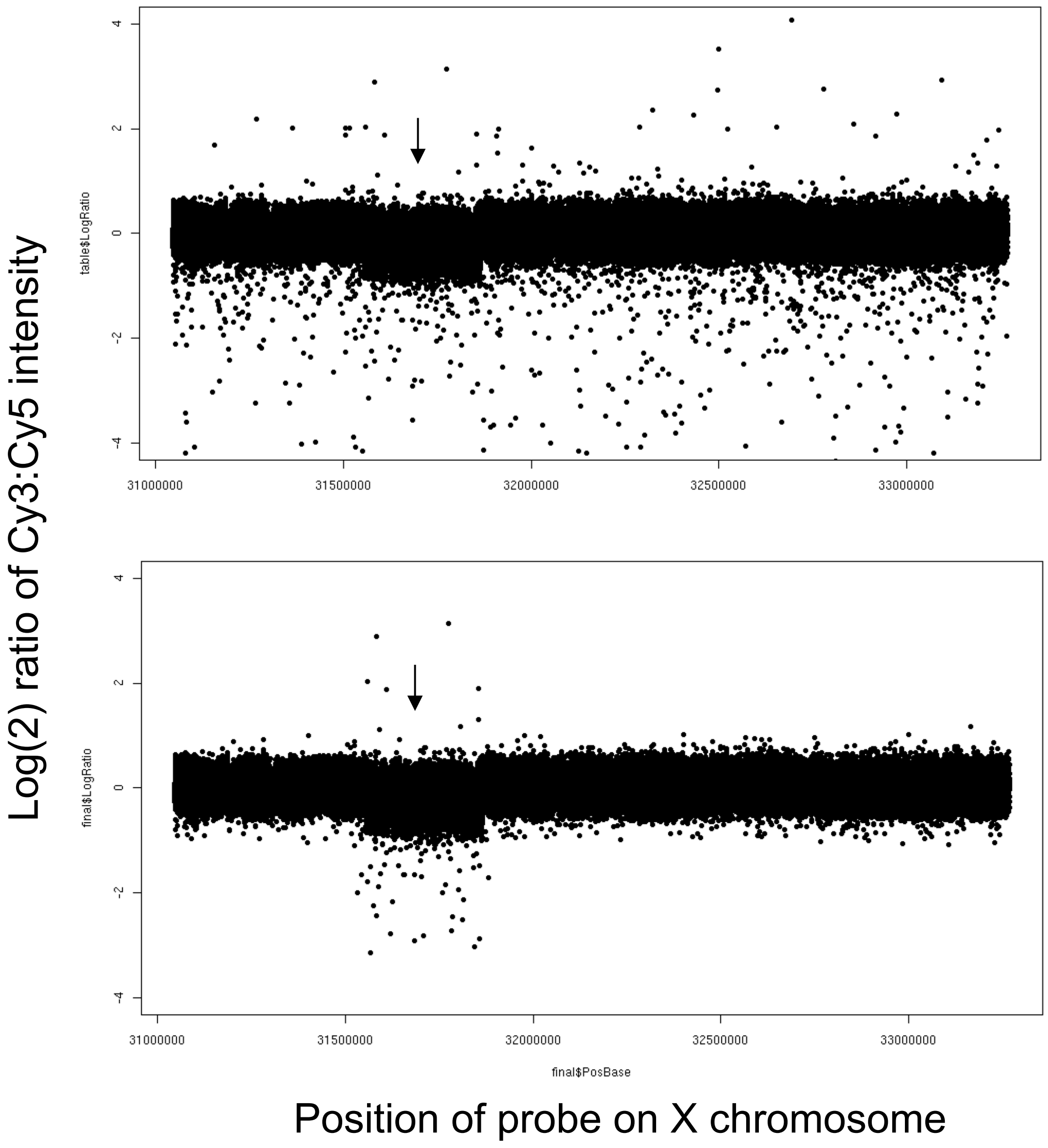

Sample 17235, all data

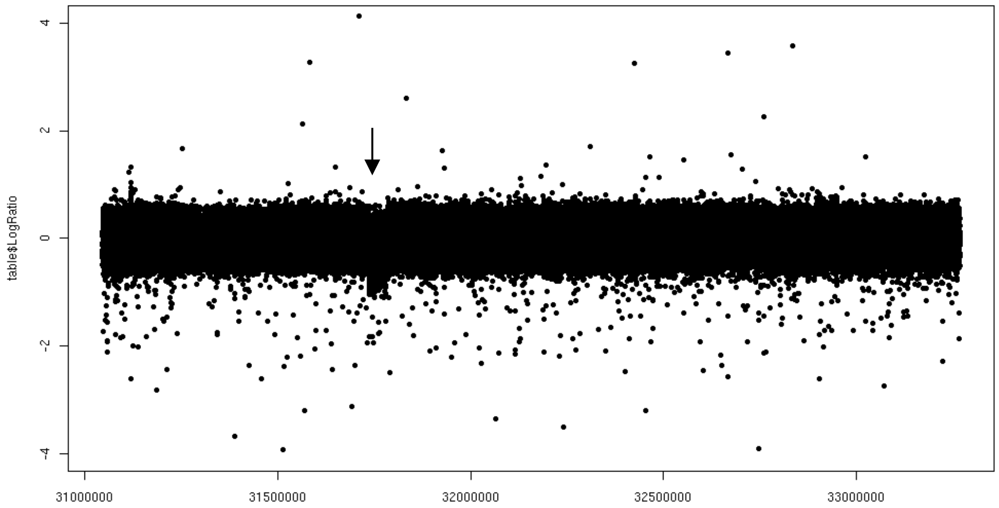

Sample 17235, high variance probes removed

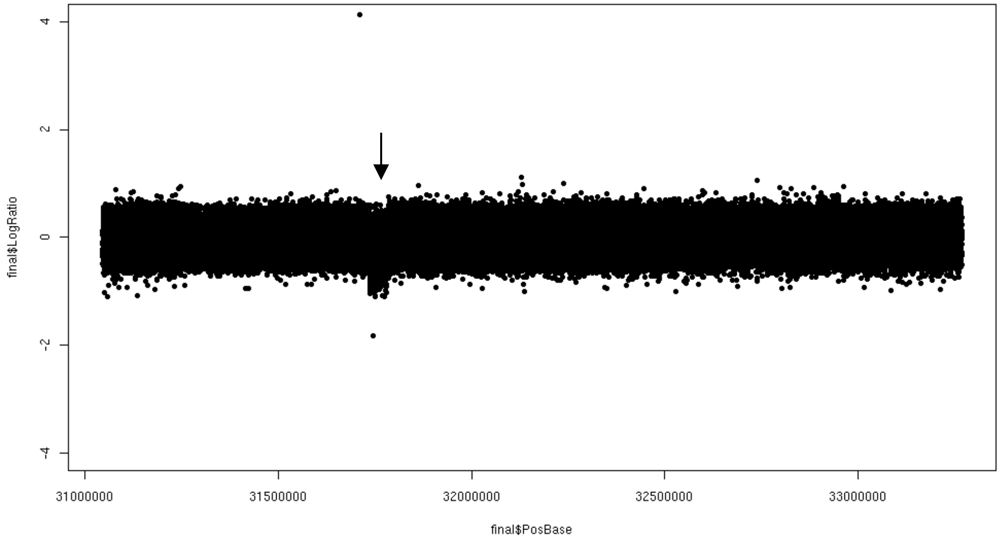

Log(2) ratio of Cy3:Cy5 intensity

Position of probe on X chromosome
